# The Psychometric Properties of the Trunk Impairment Scale in Children with Cerebral Palsy

**DOI:** 10.3390/children9030435

**Published:** 2022-03-19

**Authors:** Hyerim Jung, Young-Eun Choi

**Affiliations:** 1 Department of Occupational Therapy, Baekseok University, Cheonan 31065, Korea; hyerimhome@hanmail.net; 2 Department of Physical Therapy, College of Health Medicine, Kaya University, Gimhae 50830, Korea

**Keywords:** cerebral palsy, children, trunk impairment scale, Rasch analysis, psychometric

## Abstract

The Trunk Impairment Scale (TIS) measures static and dynamic seated trunk control in children with cerebral palsy (CP) who have postural control problems. Studies have investigated the reliability and validity of the TIS. However, the fitness and difficulty of the scale items have not been investigated. This study used Rasch analysis to test the construct validity of TIS for children with CP. TIS data were collected from 60 children with CP and analyzed for person and item fit, item difficulty, rating scale suitability, and separation reliability. Principal component analyses of residuals revealed that TIS had unidimensionality. Five misfit items (static sitting balance (SSB) items 2 and 3, dynamic sitting balance (DSB) items 4 and 5, and coordination (COO) item 3) were identified. DSB8 is the most difficult item, followed by DSB3 and COO4. On the other hand, the SSB3 item was found to be a relatively easy item. The rating scales demonstrated that out of the three subscales, SSB, DSB, and COO, only the SSB subscale did not meet the appropriate criteria. We demonstrated that statistical item analysis with the Rasch model could provide valuable information related to psychometric properties.

## 1. Introduction

Cerebral palsy (CP) is defined as motor and postural impairment owing to a nonprogressive insult to the brain of a developing fetus or postnatal infant. Postural impairment is a major characteristic of children with CP [1]. Postural control is the ability to control the position of the body in space while maintaining stability to adapt to the surrounding environment [2]. Postural control development enables sitting, reaching, standing, and walking by keeping the head and trunk upright against gravity. It also influences the development of hand–eye coordination, upper extremity function, functional skills, self-care, cognition, and social interaction [3]. However, children with CP have problems with developing postural control due to neurological symptoms such as rigidity, paralysis, reduced coordination, and sensory defects [4].

The trunk is a key segment for postural control as trunk muscles provide the foundation for spine and trunk stabilization and free movement of the head, arms, and legs [5]. In many cases, children with CP perform daily tasks while sitting instead of standing, but children with mild and severe CP exhibit postural impairment regardless of the severity of their disability [6,7]. Therefore, it is important to evaluate seated trunk control in children with CP during treatment planning and assessment [8].

Tools for measuring and assessing trunk control in children with CP include the seated postural control measure (SPCM), spinal alignment and range of motion measure (SAROMM), segmental assessment of trunk control (SATCo), sitting assessment for children with neuromotor dysfunction, and trunk control measurement scale [9]. SPCM has low reliability [10], whereas SAROMM assesses only trunk alignment and the range of motion [11]. SATCo assesses trunk control under three sitting conditions (sitting with hand support, head movement, and external perturbation) [12]. However, these tools cannot assess the static and dynamic trunk control needed to perform functional activities. The Trunk Control Measurement Scale (TCMS) is an expanded version of the TIS and was developed to measure trunk control among children with CP. It comprises three subscales: static sitting balance, selective movement control, and reaching. Evaluation using TCMS is more time-consuming than that using the TIS, and it has yet to be validated for use in children with CP under the age of 8 years [8]. Other instruments cannot assess the static and dynamic trunk control required to perform functional activities.

The TIS, designed to measure trunk control in stroke patients, was used to assess children with CP who have postural impairment by measuring static and dynamic seated trunk control [13]. A standardized assessment tool is necessary to apply evidence-based physical therapy for trunk control in children with CP to identify the degree of impairment before setting the treatment goal and intervention plan [14]. The technical adequacies of TIS and its subscales were substantiated using the classical test theory (CTT). Intraclass correlation coefficients (ICC) for inter-rater and test–retest for the total score and subscale score varied between 0.94 and 1.00. Kappa values for the items ranged from 0.45 to 1.00 [15]. The item response theory (IRT) differs from CTT in terms of item invariance. In the CTT, item discrimination is estimated based on the correlation coefficient for the item score and total score. However, the same participant is rated to have a lower ability when difficult items are used but rated to have a higher ability when easy items are used. In contrast, the IRT can objectively assess the participant’s level or ability regardless of the sample items because it considers the gap between the participant’s ability and the item’s difficulty [16]. The Rasch model is the most common IRT-based method used to assess item fit and difficulty. In other words, Rasch analysis can be used to analyze the difficulty and discrimination ability of each item [17]. Therefore, the scales that had previously been standardized using CTT are now revalidated by using IRT [18,19,20]. Hence, this study evaluated the item fit, item difficulty, and scale fit of TIS for children with CP using Rasch analysis.

## 2. Materials and Methods

### 2.1. Study Subjects

**Figure 1:**
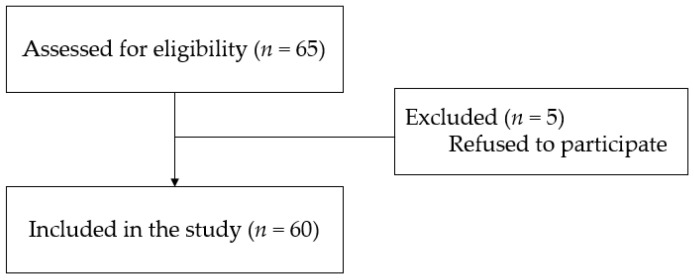
Study population.

We employed a sample of 65 children with CP who were outpatients at a hospital in Korea. The inclusion criteria were as follows: diagnosis of CP by a pediatric rehabilitation specialist, age of 6–12 years, and ability to understand and follow the therapist’s instructions [9]. The study included a convenient, nonprobabilistic sample. Five children were excluded because of the refusal to collaborate during data collection. Therefore, the final sample included 60 children with CP (26 boys and 34 girls) (Figure 1). Information about the classification and GMFCS level of each child’s CP was provided by the children’s physical therapist in the pediatric rehabilitation department. This study was conducted according to the guidelines of the Declaration of Helsinki and approved by the Institutional Review Board of Kaya University (Kaya IRB-318 and 23 April 2021). Standard deviation was used to calibrate the items for Rasch analysis. A two-tailed 99% confidence interval is ±2.6 SE wide. For a ±1 logit interval, this SE is ±1/2.6 logits. This provides a minimum sample in the range 4×(2.6)2 < *n* < 9×(2.6)2, that is, 27 < *n* < 61, depending on targeting [21]. Thus, a sample of 50 well-targeted examinees is conservative for obtaining useful, stable estimates. Thirty items administered to thirty persons (with reasonable targeting and fit) should produce statistically stable measures (±1.0 logits, 95% confidence) [22]. Sixty individuals with CP were a sufficient sample size for the analysis. Table 1 presents the general characteristics of the subjects.

### 2.2. Measurements

TIS is an assessment tool with proven reliability developed to assess balance and trunk movement coordination in a sitting position in patients who have suffered a stroke [23]. TIS consists of 17 items in three subscales: static sitting balance (SSB), dynamic sitting balance (DSB), and coordination (COO), with a total score range of 0 to 23 points. The SSB subscale contains three items: (1) the ability to maintain a sitting position with feet supported, (2) the ability to maintain a sitting position while the legs are passively crossed, and (3) the ability to maintain a sitting position when participant actively crosses the legs (Table 2). Each item is scored from 0 to 2 or 0 to 3 points for a total of 0 to 7 points. The DSB subscale contains 10 items on lateral trunk flexion and unilateral lifting of the hip. Each item is scored from 0 to 1 point for a total of 0 to 10 points. For COO, the participant is asked to rotate the upper or lower part of their trunk six times, and four items on the ability to initiate movement from the shoulder girdle or the pelvic girdle were assessed. Each item is scored from 0 to 1 or 0 to 2 points for a total of 0 to 6 points. This study used the modified Korean version of TIS [24]. Inter-rater reliability of the Korean version of TIS was ICC [3,1] = 0.920–0.983 (r = 0.924–0.984), and the test–retest reliability was ICC [3,1] = 0.805–0.901 (r = 0.806–0.903).

### 2.3. Procedure

To minimize measurement errors, the TIS measurements were performed by a single physical therapist with seven years of experience on an individual basis in a separate treatment room that was quiet and familiar to the children. During the measurement, the children were allowed to wear the shoes or braces that they usually used. The height of the mat where the children sat was set to allow the hip and knee joints to maintain a 90° angle while supporting the feet on the ground [23].

### 2.4. Data Analysis

The participants’ general characteristics were analyzed using descriptive statistics using SPSS version 26.0 (SPSS Inc., Armonk, NY, USA). Rasch analyses were performed using Winsteps 4.0.1 (Linacre, Chicago, IL, USA) to analyze item fit, item difficulty, rating scale suitability, and separation reliability. First, it was checked whether unidimensionality, the basic assumption of the Rasch model, was established according to the principal component analysis of residual for the collected data. As a result of the residual principal component analysis, if the variance explained by the Rasch measure is 50% or more and the eigenvalue of the first or second residual variance excluding the Rasch factor is less than 3.0, it can be determined that unidimensionality is supported [25]. For the item fit criteria, an item with an MnSq < 0.6 or >1.4 and a Z-value of <−2 or >2 infit index was classified as a misfit item. Item difficulty was analyzed by listing the items from the highest to lowest difficulty. The Rasch analysis rating scale model was used to analyze the suitability of the rating scale. Generally, the mean and structural measures should increase as the rating scale score increases. The fit value of each scale provides information on whether the scale is suitable or not. The fitted value cutoff for each scale was 1.0, and any scale with a fit value exceeding 1.5 was classified as ineffective [25]. Separation reliability is based on the same concept as Cronbach’s alpha, where values closer to 1 represent more ideal values. For reliability, a separation reliability coefficient of 0.70 and separation index of 1.5 were considered to be acceptable reliability, values of 0.80 and 2 indicated good reliability, and values of 0.90 and 3 indicated very good reliability [26].

## 3. Results

### 3.1. Study Participants

A total of 60 children with CP between the age of 6 and 12 years were included in this study: 10 children had unilateral palsy and 50 children had bilateral palsy (Table 1). In addition, 11, 26, 11, and 12 children had hemiparalysis, diplegia, quadriplegia, and motor dysfunction, respectively.

### 3.2. Unidimensionality

Residual principal component analysis was performed to evaluate the unidimensionality of whether the TIS, including 17 items, was suitable for the Rasch model. As a result, the variance explained by the Rasch measurement was 78.3%, and the eigenvalues of the first and second residual variances excluding the Rasch factor were 2.19 and 2.12, respectively; thus, the assumption of unidimensionality was established.

### 3.3. Fit Statistics

Table 3 shows the results of item fit of the TIS. As a result of item fit analysis, with the exception for five items, the mean square residual value of the internal fit index ranged from 0.62 to 1.02, and the Z value was between −2.0 and 2.0. The results identified five misfit items (SSB2, SSB3, DSB4, DSB5, and COO3).

### 3.4. Item Difficulty

**Figure 2:**
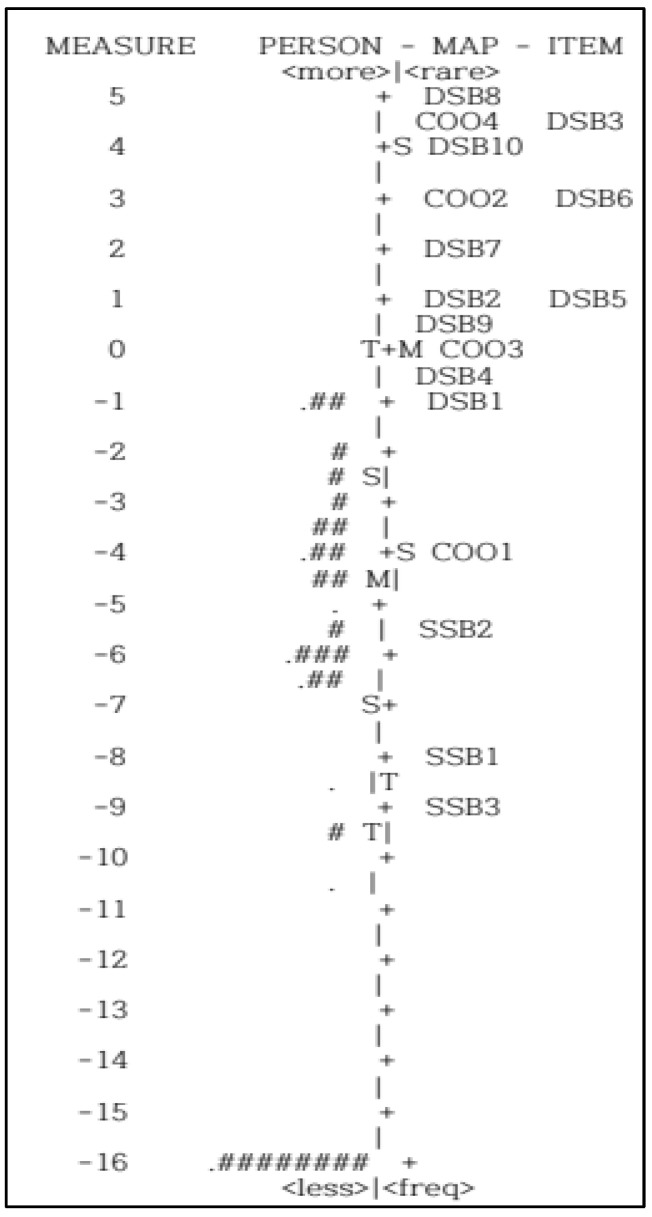
Map of personal proficiencies and item difficulties for the 17 items of the Trunk Impairment Scale. Note. More difficult items are placed on the top of the diagram. M, mean of child or item distribution; S, 1 standard deviation from the mean; T, 2 standard deviations. Each ‘#’ is two persons and each ‘∙’ is one person.

Seventeen items are listed in order of difficulty. The left side of Figure 2 shows the distribution of participants, and the right side shows the difficulty level of the items. The higher the item, the larger the logit value for a difficult item; the lower the item, the smaller the logit value is for an easier item. Therefore, DSB8 is the most difficult item, followed by DSB3 and COO4. Conversely, the SSB3 item was found to be relatively easy.

### 3.5. Suitability of the Rating Scale

As a result of analyzing the rating scales for three subscales, such as static sitting balance, dynamic sitting balance, and coordination, two subscales except the static sitting balance subscale met the appropriate criteria (Table 4). The mean square residual of the extrinsic fit index is 2.0 or less, and the mean measured values are vertically ordered. Moreover, it was analyzed to be a suitable scale category because the step-corrected value interval of each category was located between 1.0 and 5.0 logits.

### 3.6. Separation Reliability

The person separation reliability was 0.95, and the person separation index was 4.54. The item separation reliability was 0.99, and the item separation index was 9.21.

## 4. Discussion

The TIS is a tool that can measure the trunk control ability of CP and evaluate static balance ability, dynamic balance ability, and coordination in a sitting position. The TIS can evaluate children in a wide range of ages (5–19 years) with motor impairment, and it can be measured quickly and efficiently, making it useful in clinical practice. In previous studies, TIS was demonstrated to be a strong measurement tool [15,23]. The next step was to evaluate the effectiveness of TIS using Rasch analysis, which was the goal of our study. We conducted a Rasch analysis based on IRT to assess unidimensionality analysis, fit of the Rasch model for each item of TIS, item difficulty, and suitability of the rating scale.

In the Rasch analysis based on IRT, unidimensionality was maintained for children with CP, and adequate reliability and separated reliability on participants and items were observed. This suggested that TIS was suitable for measuring the trunk control of children with CP. The fit of the items further supported the unidimensionality of TIS. In the item fit analysis, all items, except for five, formed unidimensionality. The five items did not fit the unidimensionality model. Among the five items, items 4 and 5 for DSB had a mean square residual value of the outfit index less than 0.6, suggesting that the items were overfit. Such overfit items tend to overestimate the differences in the raw scores [27]. Although these two items were found to be unfit as overfit, there was no need to modify or eliminate the items from the evaluation tool since the strict item conformity analysis criteria were applied. In clinical observations, the acceptable range for the mean square residual values of the outfit index is between 0.5 and 1.7 [28]. The other unfit items, items 2 and 3 for SSB and item 3 for COO, were underfit because the mean square residual value was greater than 1.4.

Underfit items are considered to possess more changes than those predicted by the Rasch model. Items 2 and 3 for SSB measured the ability to maintain a stable sitting posture while moving the lower extremities. These activities require active trunk control such as anticipatory postural adjustment [29]. These items have a low level of difficulty and are scored highly in children who can actively control their trunk while moving their lower extremities. Item 3 for COO assessed the rotation of the lower part of the trunk. This item has a high level of difficulty and is scored highly in children who can selectively rotate their trunk in a horizontal plane. In our study, children with low ability unexpectedly received higher scores, suggesting that the item was unfit. However, this item was not eliminated since it could functionally indicate improved trunk control and provide clinically important information for intervention in children with lower abilities.

Item difficulty was analyzed by an item and person map, which transformed the ordinal scale from the logit value into the interval scale. The distribution was considered to be appropriate if the individual score and the range of difficulty distribution matched, meaning that the distribution range was similar to the range of individual ability for item difficulty in measuring the entire range of the individual’s ability [30]. Among 60 participants, nine (15%) deviated from the range of item difficulty, demonstrating that their abilities were lower than the level of item difficulty. If the balance using TIS is evaluated in clinical practices, trunk impairment of children with mild motor impairment can be more accurately evaluated.

The following conditions must be met to satisfy the requirements of a rating scale in a Rasch analysis; the number of responses in each category must be ≥10, the observed averages of scores must be arranged from low to high, structural estimates must show intervals with a difference of at least 1.4 but no more than 5 logits for a clear division between categories, the vertices must be visually distinguishable in the probability curve, and the outfit index MnSq of each rating scale must be ≤2 [31]. In this study, all subscales from the TIS met the requirements except the 3-point scale of SSB. In SSB, the score of 1 point did not meet the requirements where the number of responses should be at least 10 and the structural estimates among each category should be increased by an increase of scale. However, the scores of 1 point and 2 points showed a reverse effect where structural estimates declined as the scale increased. Step calibration disordering indicated that the rating scale had a poor function and suggested the need for expansion, reduction, or modification of the rating category. However, this phenomenon may be observed when there are transition categories. Transition categories are narrow intervals of latent variables and were important factors in developing the rating scale [32]. Transition categories indicate the transition between the dominant categories and are not observed more than neighboring dominant categories. Thus, the probability of observing a transitional category tends to be low. Item 3 for SSB evaluated the active crossing of the unaffected leg over the affected leg in a sitting position. A score of 0, 1, 2, or 3 was given for falling, unable to perform without arm support, successful crossing of the legs with the body leaned backward, or the use of hands and crossing the legs without leaning back or using hands, respectively. If scores of 1 point and 0 points were given for success and failure, respectively, cases that were close to success and failure were omitted. However, each category can be identified by distinguishing the level of active trunk control required to improve a stable posture during lower extremity movements. Therefore, rather than attempting to eliminate transition categories, collecting additional data from children with a low performance level in order to further evaluate the function of the TIS is recommended.

This study had a few limitations. First, since our study comprised 60 children with CP in hospitals with an outpatient setting and used TIS to perform analysis, our study results cannot be generalized. To generalize the results, further studies are required with a larger sample size to enhance its statistical power and probability. Second, this study included only children aged 6–12 years old with CP. This may prevent the generalization of the results onto children with CP of all ages. Further studies are necessary for teens with CP. Lastly, different item functions were not investigated in this study. Since there might be items that function differently by the type of CP, future studies need to evaluate the differences in trunk control according to the type of CP as well. However, this study is meaningful since the Rasch model was used to investigate TIS and provided additional information on the psychometric properties of TIS and more psychometric evidence of TIS in assessing the trunk control in children with CP.

## Figures and Tables

**Table 1:** General characteristics of the study participants (*n* = 60).

Classification	Frequency	Mean Age (years)	Age Range	Unilateral (*n*)	Bilateral (*n*)	Sex	
						Boy (*n*)	Girl (*n*)
GMFCS level I	10	8.0	6–11	3	8	3	8
GMFCS level II	14	8.4	6–12	5	9	7	7
GMFCS level III	12	8.9	6–12	3	9	7	5
GMFCS level IV	12	9.0	6–12	0	12	4	8
GMFCS level V	11	9.2	7–11	0	11	5	6

GMFCS, gross motor function classification system.

**Table 2:** Summary of the items of the Trunk Impairment Scale (TIS).

Item	Description
Static sitting balance	
1	Keep sitting balance
2	Keep sitting balance with legs crossed
3	Keep sitting balance while crossing legs
Dynamic sitting balance	
1	Touch seat with elbow, most affected side
2	Touch seat with elbow, most affected side (repeat item 1, trunk movement)
3	Touch seat with elbow, most affected side (repeat item 1, compensation strategies)
4	Touch seat with elbow, less affected side
5	Touch seat with elbow, less affected side (repeat item 4, trunk movement)
6	Touch seat with elbow, less affected side (repeat item 4, compensation strategies)
7	Lift pelvis from seat, most affected side
8	Lift pelvis from seat, most affected side (repeat item 7, compensation strategies)
9	Lift pelvis from seat, less affected side
10	Lift pelvis from seat, less affected side (repeat item 9, compensation strategies)
Coordination	
1	Rotate shoulder girdle 6 times
2	Rotate shoulder girdle 6 times within 6 s
3	Rotate pelvis girdle 6 times
4	Rotate pelvis girdle 6 times within 6 s

**Table 3:** Item fit statistics.

Item	Logit	SE	Infit		Outfit	
			MnSq	Z-Value	MnSq	Z-Value
SSB1	−8.11	0.45	0.69	−1.12	0.44	−1.46
SSB2 *	−5.46	0.40	2.09	3.65	1.82	1.81
SSB3 *	−9.23	0.42	2.14	3.63	3.38	3.62
DSB1	−1.19	0.39	0.87	−0.49	0.92	−0.14
DSB2	1.20	0.39	0.95	−0.17	0.86	−0.17
DSB3	4.26	0.47	0.74	−0.98	0.48	−0.54
DSB4 *	−0.59	0.39	0.51	−2.49	0.38	−2.29
DSB5 *	1.05	0.39	0.54	−2.39	0.40	−1.61
DSB6	3.10	0.42	0.74	−1.20	0.46	−0.64
DSB7	2.12	0.40	0.78	−1.01	0.55	−0.65
DSB8	5.00	0.53	0.50	−1.93	0.21	−0.86
DSB9	0.60	0.39	0.68	−1.56	0.54	−1.27
DSB10	3.84	0.45	0.90	−0.31	0.57	−0.41
COO1	−3.95	0.38	1.02	0.16	0.85	−0.37
COO2	3.10	0.42	0.88	−0.49	0.59	−0.38
COO3 *	0.01	0.39	1.76	2.86	1.78	1.87
COO4	4.26	0.47	0.62	−1.54	0.34	−0.88

SSB, static sitting balance; DSB, dynamic sitting balance; COO, coordination; MnSq, mean square. * Misfit item.

**Table 4:** Summary of the rating scale analysis of the TIS.

Subscale	Category Level	Observed Count	Observed Average	Infit	Outfit	Step Calibration
				MnSq	MnSq	
Static sitting balance	0	59	−5.79	1.08	3.12	None
	1	5	−3.04	1.48	0.97	−4.62
	2	103	2.92	1.28	0.96	−4.80
	3	13	9.78	0.58	0.15	9.42
Dynamic sitting balance	0	370	−2.86	0.99	0.89	None
	1	230	2.51	0.99	0.61	1.02
Coordination	0	149	−6.66	1.20	1.32	None
	1	61	0.48	0.96	0.39	−3.27
	2	30	5.82	0.86	0.75	3.27

## Data Availability

The data presented in this study will be provided upon request.
